# Metabolomic signatures of tuberculosis and paratuberculosis in goats revealed by high-resolution and benchtop NMR spectroscopy

**DOI:** 10.1186/s13567-025-01686-7

**Published:** 2025-12-12

**Authors:** Pilar Alonso-Moreno, Paula Ortiz, Javier Ortega, Carlos Velasco, Alba López, Javier Bezos, Jose Luis Izquierdo-Garcia

**Affiliations:** 1 NMR and Imaging in Biomedicine Group, Pluridisciplinary Institute, Complutense University of Madrid, Madrid, Spain; 2 Department of Chemistry in Pharmaceutical Sciences, Pharmacy School, Complutense University of Madrid, Madrid, Spain; 3 VISAVET Health Surveillance Centre, Complutense University of Madrid, Madrid, Spain; 4 Department of Animal Health, Faculty of Veterinary Medicine, Complutense University of Madrid, Madrid, Spain; 5 ICTS Bioimagen Complutense, BIOIMAC, Complutense University of Madrid, Madrid, Spain; 6 CIBER de Enfermedades Respiratorias (CIBERES), Instituto de Salud Carlos III, Madrid, Spain

**Keywords:** Tuberculosis, nuclear magnetic resonance, benchtop, goats, metabolomics

## Abstract

Goats represent a significant reservoir for tuberculosis (TB) in animals, contributing notably to public and animal health challenges, causing economic repercussions. Ante mortem diagnosis of TB is hindered by the limited sensitivity of available techniques and false-positive results from other mycobacterial infections, such as paratuberculosis (PTB). Nuclear Magnetic Resonance (NMR)-based metabolomics provides unique fingerprinting of the disease’s metabolic status, making it a promising diagnostic tool. However, conventional high-resolution NMR has limitations in veterinary practice, where high costs and large equipment size are major constraints. Benchtop NMR spectrometer is proposed as a compact, cost-effective alternative for livestock farms. The study aimed to evaluate NMR-based metabolomics as a diagnostic tool and transfer it from high-resolution to benchtop NMR spectrometers in an animal setting. Serum samples from TB-infected, PTB-infected (*n* = 16), and healthy control goats (HC) were analyzed by both high-resolution and benchtop NMR spectroscopy. Multivariate statistical analysis successfully differentiated groups on the basis of their metabolic profiles with both spectrometers. We identified that betaine, glucose, glycerol, and lactate are significantly capable of distinguishing between the three groups. Additionally, 3-hydroxybutyrate, creatine, glutamate, leucine, lysine, phenylalanine, threonine, and tyrosine further differentiate TB from HC. Acetate, creatine, glutamate, isoleucine, leucine, and lysine distinguish TB from PTB, while 3-hydroxybutyrate and phenylalanine serve to differentiate PTB from HC. Analyses with both high-resolution and benchtop spectrometers demonstrated high sensitivity and accuracy and reliable metabolite identification. These findings highlight NMR’s spectroscopy potential to identify biomarkers associated with TB and PTB infection, improving health management in livestock.

## Introduction

Tuberculosis (TB) is a zoonotic disease that represents a significant global health challenge, with profound impacts on both public health and economic systems worldwide [1, 2]. This disease in animals, caused by species within the *Mycobacterium tuberculosis* complex, includes prominent members such as *Mycobacterium caprae* and *Mycobacterium bovis*, which predominantly affect livestock [2–4]. These zoonotic pathogens complicate eradication efforts and pose serious risks to animal health and food security. In particular, TB in goats and cattle remains a major concern and is targeted by national eradication programs within the European Union, mainly owing to its impact both on animal and public health and welfare and on productivity indexes [5, 6].

Ante mortem diagnosis of TB in goats is mainly based on the single intradermal tuberculin (SIT) and the comparative intradermal tuberculin (CIT) tests. However, despite their cost-effectiveness, these techniques present limitations, including technical issues related to the in vivo antigenic stimulation of the animals and the need for two visits to the farm, the subjective component in result interpretation, and the limited performance in terms of sensitivity and specificity [7, 8]. In certain circumstances, the in vitro interferon-gamma release assay (IGRA) is also used for TB diagnosis in goats, offering, in general terms, a higher sensitivity in comparison with SIT/CIT tests without an in vivo previous antigenic stimulation. However, IGRA has a higher cost and logistical requirements, and limited specificity under specific epidemiological situations [7, 9]. Moreover, in goats, both diagnostic methods also face the limitation of potential false-positive results due to infection by non-tuberculous mycobacteria such as *Mycobacterium avium* subspecies *paratuberculosis* (MAP) [7, 10], the causal agent of paratuberculosis (PTB). PTB, known as Johne’s disease, is a chronic granulomatous enteritis that predominantly affects young ruminants owing to ingestion of contaminated food or water from the environment [11, 12]. Vaccination against PTB in Spain is allowed in goats and may cause diagnostic interferences on the most sensitive diagnostic tests (SIT test and IGRA) [13].

Owing to these limitations, the detection of infected animals is affected, making the eradication of the disease more difficult. Therefore, better novel ante mortem diagnostic methodologies are needed. Advances in omics sciences, particularly metabolomics, offer promising avenues for early disease detection [10, 14, 15]. Metabolomics involves the comprehensive study of small molecular weight metabolites (< 1,500 Da), which are key indicators of physiological and pathological processes in living organisms. These metabolites can serve as sensitive biomarkers for detecting perturbations in biological pathways, allowing for earlier diagnosis compared with traditional methods.

Nuclear magnetic resonance (NMR) spectroscopy is one of the principal techniques used in metabolomics. NMR is highly robust and reproducible and requires minimal sample preparation, making it suitable for biomedical applications [16–18]. High-resolution (HR)-NMR has demonstrated substantial potential for both diagnosis and prognosis applications in disease research [19–21]. Among the available instruments, 600 MHz NMR spectrometers are widely regarded as the gold standard for metabolomic analysis. These systems are equipped with a superconducting magnet housed withing two cryogenic dewars (one containing liquid helium and the other liquid nitrogen), which enable the generation of the strong magnetic fields required for high-resolution NMR. Owing to their high sensitivity and specificity, these instruments can detect approximately 30–50 metabolites in a serum sample within 10–20 min per proton NMR experiment [19]. However, these instruments are large and costly, in terms of both acquisition (approximately €700 000 to €2 million) and maintenance (up to €70 000€ per year, owing to the need for cryogenic liquids to sustain the superconducting magnet). Moreover, their operation requires specialized expertise, which limits their widespread adoption in routine biomedical or veterinary settings. In recent years, benchtop NMR (b-NMR) spectrometers have emerged as an accessible and cost-effective alternative, offering compact size, portability, and sufficient analytical power for many applications [22]. These instruments rely on permanent magnet technology, which substantially reduces acquisition costs (around €70 000–120 000) and virtually eliminates maintenance expenses, as no cryogenics liquids are required. Additionally, their user-friendly operation makes them suitable for broader implementation. While benchtop NMR operates at lower magnetic fields—resulting in decreased resolution since signal intensity scales with the magnetic field strength (*B*_0_^3/2^) and specificity depends on the Larmor frequency—these systems still provide valuable metabolic information. Typical acquisition times range from 15 to 30 min per sample, enabling the detection of approximately 19 metabolites in serum [22]. Although the lower sensitivity and longer acquisition times are limitations, these can be offset in biomedical and veterinary contexts by deploying multiple instruments in parallel or integrating them into high-throughput analytical pipelines.

Previous studies have demonstrated the potential of metabolomics, particularly through b-NMR, in differentiating metabolic profiles associated with TB and PTB in cattle [10] and TB in humans [20, 21]. Building on this expertise, the present study aims to deepen our understanding of the metabolic mechanisms underlying TB in goats and to establish the diagnostic capabilities of benchtop NMR as a practical tool for veterinary applications. By advancing NMR-based metabolomics for use with portable, cost-effective instruments, we seek to enhance the detection and monitoring of TB in livestock, ultimately contributing to improved animal health and broader public health efforts.

## Materials and methods

### Animal selection

Sixty-seven serum samples from goats were analyzed. Samples were provided by the Veterinary Health Surveillance Centre (VISAVET) at Complutense University of Madrid, Spain. The samples were collected from two farms located in the Community of Madrid and Castilla and León (Ávila province). These samples were divided into three groups: TB-infected, PTB-infected, and healthy controls (HC).

The TB group (*n* = 26) included goats from a TB-infected herd. These goats belong to a Guadarrama breed herd located in the Community of Madrid with a previous history of TB infection confirmed by bacteriological culture (*M. bovis*; SB0121). Goats from this group were reactor both IGRA (Bovigam TB kit, Thermo Fisher Scientific, USA) and SIT test. These tests were applied in TB the context of control programs to detect pathogen-specific immune responses.

The PTB group (*n* = 16) consisted of goats from a TB-free herd infected with *Mycobacterium avium* subspecies *paratuberculosis* (MAP), a pathogen that may cause clinical signs and interfere with the diagnosis of TB, although it is not subjected to compulsory control programs. Goats of this group were Murciano-Granadina breed form a herd located in Castilla and León. The herd had no previous history of TB in the last 7 years. Also, the herd had never been subjected to a vaccination program against PTB, and the presence of MAP in the herd was confirmed using a polymerase chain reaction (PCR) for environmental DNA sampling using GPSponges kit (Genetic PCR Solutions, Spain) [23]. Positive PTB status of these goats was defined using a commercial Paracheck kit (Thermo Fisher Scientific, USA).

The HC group consisted of goats (*n* = 25) from a herd with a TB and PTB-free history of infection. Individual status of the animals was verified through the previously commented diagnostic tests for both diseases.

### Sample processing

Blood samples were collected from all the animals in the context of the control programs by trained veterinary staff through jugular venipuncture, into tubes with no additives. Then, the blood samples were centrifuged (1500 × *g* for 10 min), and serum was collected. Afterwards, serum samples were centrifuged at 15 000 × *g* at 4 °C using Amicon Ultra-0.5 3000 MWCO filters to remove protein or lipid components that could interfere with the metabolic profile analysis [10]. A total of 300 μL of filtered serum was mixed with 300 μL of deuterated water containing 1 mM trimethylsilylpropanoic acid (TSP), which served as the internal standard for metabolic quantification. The use of internal buffers maintained a stable pH, resulting in minimal signal drift. For certain samples, adjustments were made to the filtered volumes, and the amount of deuterated water with TSP was modified accordingly to maintain a consistent final concentration.

### High-resolution NMR spectroscopy 

Serum samples were analyzed by high-resolution NMR spectroscopy using a Bruker AVIII 700 MHz spectrometer (Complutense University of Madrid, Spain) operating at a frequency of 700.17 MHz.

One-dimensional ^1^H-NMR spectra were acquired at 4 °C employed a presaturated Nuclear Overhauser Effect SpectroscopY (NOESY) experiment to suppress the water signal. Standard solvent suppress spectra were acquired into 32,768k data points, averaged over 256 scans or acquisitions. A 16.08 ppm sweep width, a 1.45 s acquisition time, a 150 ms mixing time, a 2 ms recycle delay, and a 2 ms saturation delay were used. A spectral width of 1126.26 Hz was employed. Prior to Fourier transformation, the data were zero-filled, and free induction decays (FIDs) were multiplied by exponential line broadening factor of 1 Hz.

### Benchtop-NMR spectroscopy

The same serum samples previously analyzed by HR-NMR were subsequently evaluated using a Magritek Spinsolve 80 MHz ULTRA (1.4 T; Complutense University of Madrid, Spain), operating at a frequency of 80.18 MHz. One-dimensional ^1^H-NMR spectra were acquired at 25 °C using a one-dimensional (1D) water suppression enhanced through T_1_ relaxation (WET SUP) pulse sequence to suppress the water signal. The acquisition parameters were set to 128 scans, an acquisition time of 3.2 s, and a repetition time of 15 s. Shimming was performed before each acquisition to ensure optimal signal quality.

### Spectral processing

Spectral processing was carried out using MestReNova software (version 15; Mestrelab Research S.L., Santiago de Compostela, Spain), following established protocols [11, 23, 24]. To mitigate the random variations in water resonance suppression, the chemical shift regions from 4.65 to 4.95 ppm in b-NMR and from 4.9 to 5.05 ppm in HR-NMR were excluded. The region around 0.00 ppm, which contained the internal reference (TSP), was aligned to standardize metabolite signals but was later excluded from the statistical analysis (from 0 to 0.04 ppm). Spectral phasing was manually adjusted, and baseline correction was applied using the Whittaker Smoother algorithm [24].

### Analysis of metabolomic data

Prior to statistical analysis, the spectral data were processed to ensure consistency across samples. The spectra were divided into integrated segments, or bins, of uniform length (0.001 ppm) to account for minor shifts in resonance positions (binning) [20]. Binning was not necessary for b-NMR owing to its lower resolution and optimal sample preparation. All spectral bins were normalized on the basis of the total spectral area [25], and Pareto scaling was applied to enhance comparability across samples [26].

Multivariate statistical analysis of the ^1^H-NMR spectra was conducted using MetaboAnalyst version 6.0 software [27]. To explore patterns in the dataset, Principal Component Analysis (PCA) was employed as an unsupervised method to reduce dimensionality by identifying principal components (PCs) that explain the largest variance in the data [28]. PC1 captures most of the variation in the spectra, while subsequent components (e.g., PC2) describe additional orthogonal sources of variation.

For group categorization, a supervised model, Partial Least-Squares Discriminant Analysis (PLS-DA), was utilized to maximize the covariance between spectral data and class labels [29]. The optimal number of PLS components for the predictive model was determined using *R*^2^ and *Q*^2^ values, calculated via fivefold cross-validation [30]. In addition to class discrimination, PLS-DA identified the spectral regions most important for distinguishing between TB-infected goats and controls, based on Variable Importance in Projection (VIP) scores [20].

To evaluate the performance and reliability of the PLS-DA classification model, a Receiver Operating Characteristic (ROC) curve analysis was performed for both spectrometers. The number of components included in each classification model was determined according to the *Q*^2^ and *R*^2^ parameters obtained from the exploratory PLS models, ensuring optimal predictive and descriptive power. Model robustness and predictive accuracy were further validated through *k*-fold cross-validation, and the Area Under the Curve (AUC) was calculated as an overall indicator of classification performance [31].

### Metabolite assignments

The software Chenomx NMR Suite 9.0 (Chenomx Inc., Edmonton, Canada) was used for the assignment and quantification of metabolic signals detected in the ^1^H-NMR spectra [32]. Only metabolites with a Variable Importance in Projection (VIP) score greater than 2 from the PLS-DA analysis were selected for quantification and subsequent statistical evaluation. Differences in metabolite concentrations between groups were assessed using Student’s *t*-test, assuming unequal variances, with a significance threshold of *p*-value < 0.05 [33].

### Metabolic pathway analyses

The quantification of metabolites indicative of metabolic profile alterations enabled their integration into a pathway analysis to identify perturbed pathways and their biological relevance. Pathway analyses were conducted using MetaboAnalyst 6.0 software. This analysis incorporated both functional enrichment analysis and pathway topology analysis [27, 34, 35]. For the analysis, the *Capra hircus* (goat, KEGG) metabolomic pathway from the Kyoto Encyclopedia of Genes and Genomes (KEGG) was utilized, and GlobalAncova was selected as the method for pathway enrichment analysis [36, 37].

## Results

### Multivariate statistical analysis

**Figure 1:**
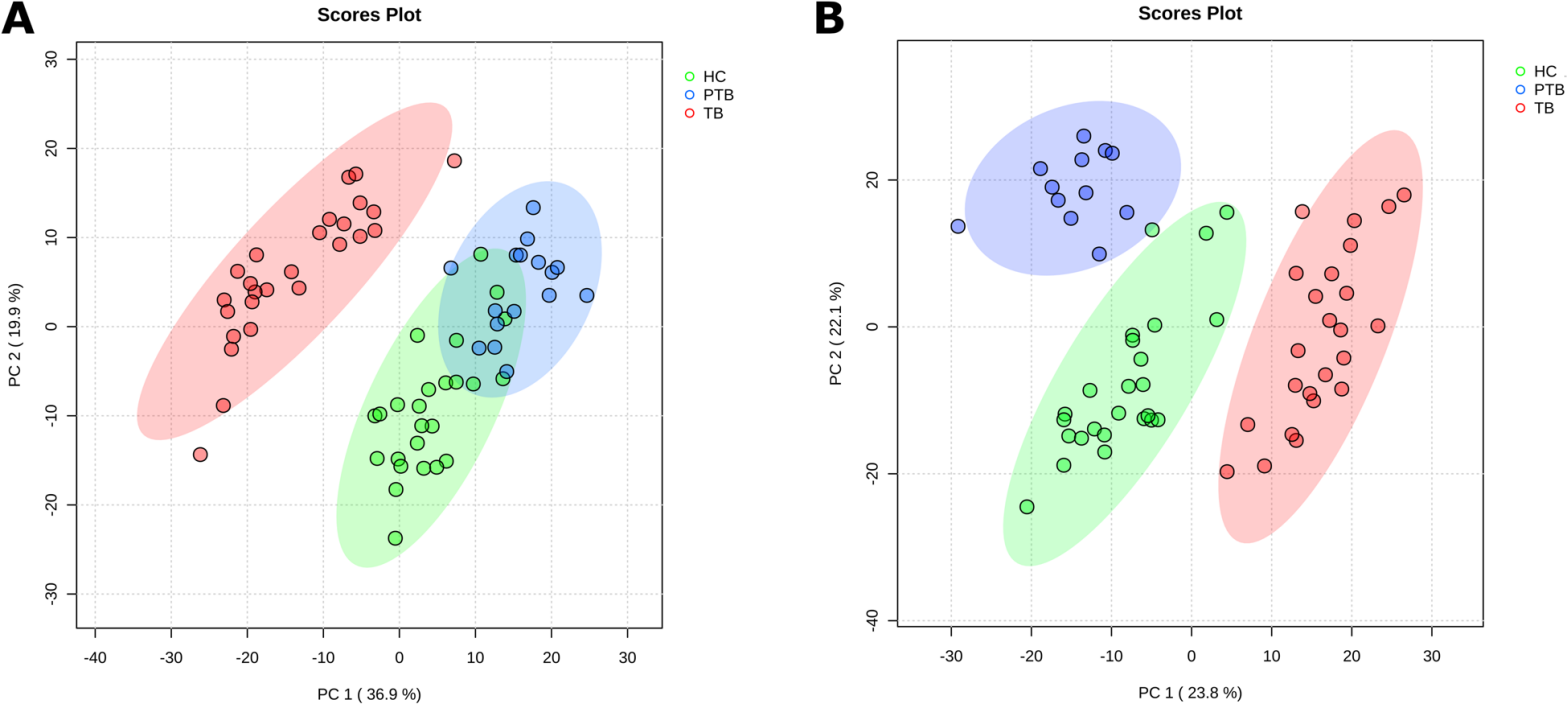
**Principal Component Analysis (PCA) score plots of serum spectra from study groups (TB, PTB, and HC goats) analyzed by** (**A**) **high-resolution and** (**B**) **benchtop NMR spectroscopy**. PC: principal component. TB: tuberculosis infection. PTB: paratuberculosis infection. HC: healthy control.

Unsupervised PCA revealed distinct metabolic patterns among the groups in both high-resolution and benchtop NMR spectra. In the HR-NMR data, PCA showed a clear separation of the groups (Figure 1A). The first principal component (PC1), which accounted for 36.9% of the variance, primarily differentiated the TB group from HC and PTB, while the second component (PC2, 19.9%) separated PTB from HC. Similarly, in the b-NMR analysis, PCA also identified distinct metabolic patterns between the groups (Figure 1B). PC1 accounted for 23.8% of the variance and drove the primary separation. Four outliers (one TB and three PTB samples) were excluded from the analysis owing to dilution effects during sample preparation.

**Figure 2:**
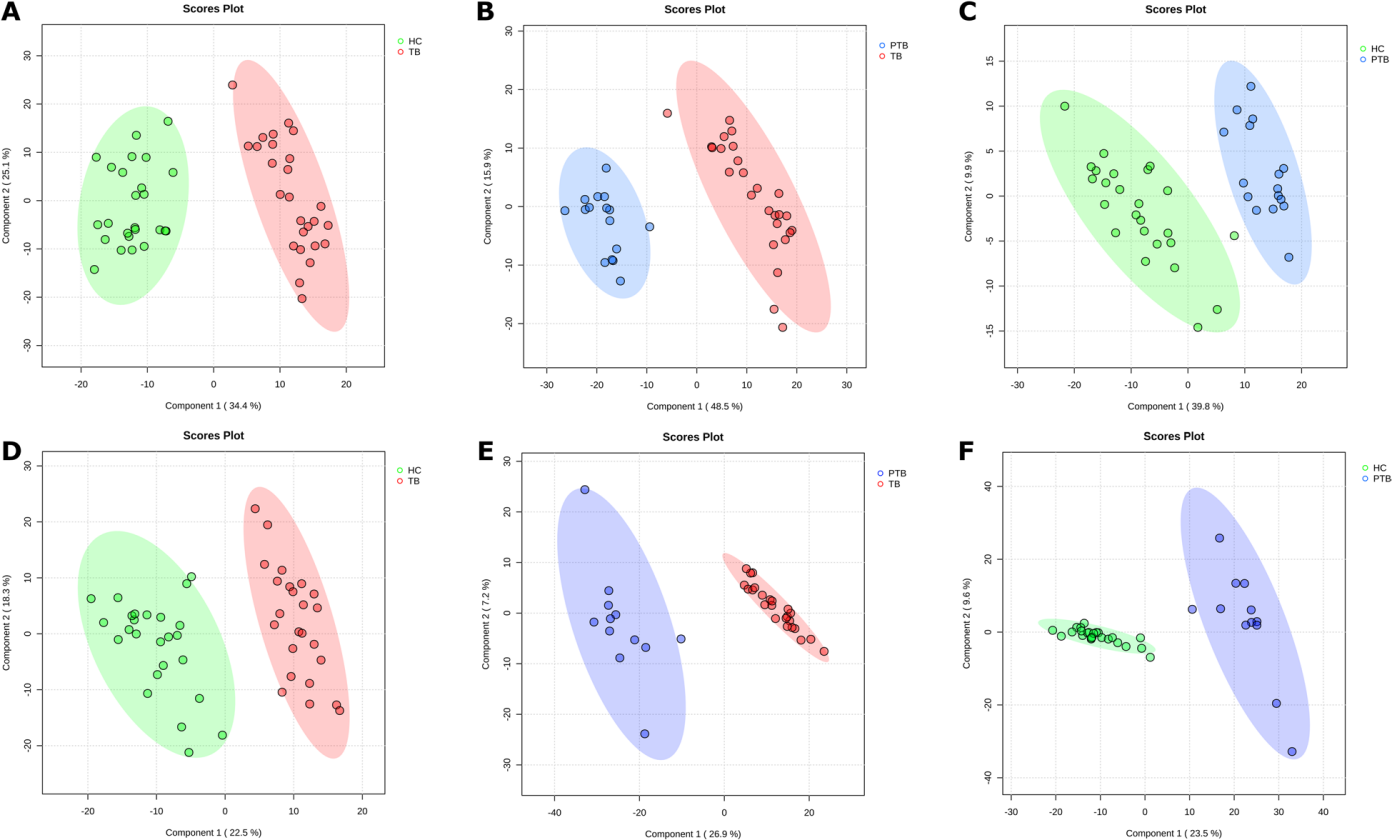
**Partial Least-Squares Discriminant Analysis (PLS-DA) score plots of serum spectra**. **A** TB versus HC, **B** TB versus PTB, and **C** PTB versus HC acquired by high-resolution NMR, and **D** TB versus HC, **E** TB versus PTB, and **F** PTB versus HC acquired by benchtop NMR spectroscopy. TB: tuberculosis infection. PTB: paratuberculosis infection. HC: healthy control.

Supervised PLS-DA successfully classified the samples into the three groups in both HR-NMR and b-NMR data. For HR-NMR, component 1 effectively differentiated between TB and HC (34.4%, Figure 2A), TB and PTB (48.5%, Figure 2B), and PTB and HC (39.2%, Figure 2C). The model demonstrated strong predictive power with fivefold cross-validation, yielding an accuracy of 100% for differentiating TB from HC (*R*^2^ = 0.933, *Q*^2^ = 0.917), 100% for TB versus PTB (*R*^2^ = 0.995, *Q*^2^ = 0.957), and 97.5% for PTB versus HC (*R*^2^ = 0.985, *Q*^2^ = 0.924). The b-NMR data also produced robust PLS-DA models, effectively separating the groups. Component 1 distinguished TB from HC (22.5%, Figure 2D), TB from PTB (26.9%, Figure 2E), and PTB from HC (23.5%, Figure 2F). Model validation via fivefold cross-validation confirmed high accuracy, with 98% for TB versus HC (*R*^2^ = 0.862, *Q*^2^ = 0.842), 100% for TB versus PTB (*R*^2^ = 0.991, *Q*^2^ = 0.962), and 100% for PTB versus HC (*R*^2^ = 0.996, *Q*^2^ = 0.937).

These multivariate statistical approaches not only enabled well-defined differentiation between TB, PTB, and HC but also highlighted the metabolic distinctions that underline these separations. The next step involved the identification of the key spectral regions responsible for these group-specific metabolic profiles.

### Biomarker identification

PLS-DA analysis further revealed the most critical spectral regions for group differentiation, as indicated by the VIP scores with both spectrometers (HR-NMR and b-NMR). In HR-NMR spectra (Table 1), 36 significant spectral regions associated with 12 metabolites were identified for the classification between TB and HC, including 3-hydroxybutyrate, betaine, creatine, glucose, glutamate, glycerol, lactate, leucine, lysine, phenylalanine, threonine, and tyrosine. For the TB versus PTB classification, 42 regions were linked to 10 metabolites, including acetate, betaine, creatine, glucose, glutamate, glycerol, isoleucine, lactate, leucine, and lysine. Lastly, PTB versus HC revealed 20 regions corresponding to 6 metabolites, such as 3-hydroxybutyrate, betaine, glycerol, glucose, lactate, and phenylalanine.

**Table 1:** **Relative intensity of the identified metabolites by HR-NMR were compared between groups with tuberculosis (TB), paratuberculosis (PTB), and healthy controls (HC)**.

	TB		PTB		HC		Student’s *t*-test (*p*-value)		
Metabolite	Mean	SD	Mean	SD	Mean	SD	TB versus HC	TB versus PTB	PTB versus HC
3-Hydroxybutyrate	2.96	1.28	0.85	0.32	4.17	1.82	< 0.001	< 0.0001	< 0.0001
Acetate	3.85	2.30	0.20	0.16	2.48	2.49	0.0653	< 0.0001	< 0.01
Betaine	6.81	1.98	2.33	0.65	2.62	0.73	< 0.0001	< 0.0001	0.1502
Creatine	3.19	0.68	1.69	0.43	2.39	0.61	< 0.0001	< 0.0001	< 0.01
Creatinine	4.60	1.56	1.60	0.59	2.63	0.90	< 0.0001	< 0.0001	< 0.01
Glucose	7.88	1.89	1.81	0.97	2.07	1.30	< 0.0001	< 0.0001	0.4766
Glutamate	7.18	2.33	2.43	0.35	3.06	0.85	< 0.0001	< 0.0001	< 0.01
Glycerol	29.76	11.75	82.03	12.94	36.98	12.93	0.0731	< 0.0001	< 0.0001
Lactate	4.38	1.10	12.09	2.21	12.69	2.22	< 0.0001	< 0.0001	0.2674
Leucine	3.48	0.70	2.16	0.67	2.61	0.83	< 0.001	< 0.0001	0.0745
Lysine	3.33	0.79	2.80	0.54	2.14	0.21	< 0.0001	< 0.05	< 0.0001
Phenylalanine	10.98	4.32	3.48	1.85	8.23	4.70	0.0532	< 0.0001	< 0.01
Threonine	18.38	5.39	54.01	11.83	39.86	10.53	< 0.0001	< 0.0001	< 0.01
Tyrosine	5.96	1.89	3.80	0.78	4.05	0.69	< 0.0001	< 0.0001	0.2415

Statistical significance was assessed using Student’s -test, with significance set at *p* < 0.05. SD: standard error.

Similarly, b-NMR analysis highlighted key metabolic differences, identifying 37 significant chemical shifts related to 6 metabolites (Table 2), including creatinine, isoleucine, glucose, glycerol, lactate, and threonine, for TB versus HC classification and fewer but distinct shifts for TB versus PTB and PTB versus HC. For TB versus PTB classification, 22 regions corresponding with glucose, glycerol, and isoleucine. Finally, in the PTB versus HC classification, 19 regions were linked to 3-hydroxybutyrate, betaine, creatinine, glucose, glycerol, and lactate.

**Table 2:** **Relative intensity of the identified metabolites by b-NMR compared between groups with tuberculosis (TB), paratuberculosis (PTB), and healthy controls (HC)**.

	TB		PTB		HC		Student’s *t*-test (*p*-value)		
Metabolite	Mean	SD	Mean	SD	Mean	SD	TB versus HC	TB versus PTB	PTB versus HC
3-Hydroxybutyrate	0.42	0.11	0.26	0.19	0.99	0.26	< 0.001	< 0.01	< 0.001
Betaine	1.44	0.35	0.92	0.38	1.64	0.59	0.171	< 0.001	< 0.001
Creatinine	0.27	0.08	0.52	0.29	0.76	0.21	< 0.001	< 0.001	< 0.01
Glucose	0.52	0.1	0.11	0.15	0.21	0.07	< 0.001	< 0.001	< 0.01
Glycerol	1.54	0.61	2.93	0.69	1.62	0.61	0.6137	< 0.001	< 0.001
Leucine	1.74	0.42	2.37	0.59	4.37	0.96	< 0.001	< 0.001	< 0.001
Lactate	0.36	0.08	0.38	0.19	1	0.22	< 0.001	0.5683	< 0.001
Threonine	0.57	0.17	0.63	0.37	1.16	0.33	< 0.001	0.5166	< 0.001

Statistical significance was assessed using Student’s -test, with significance set at *p* < 0.05. SD: standard error.

### Validation of the diagnostic models

The diagnostic PLS-DA models, constructed using two components, were evaluated through ROC curve analysis. The resulting AUC values demonstrated excellent discriminative performance for both spectrometers, confirming the robustness of the classification approach.

**Figure 3:**
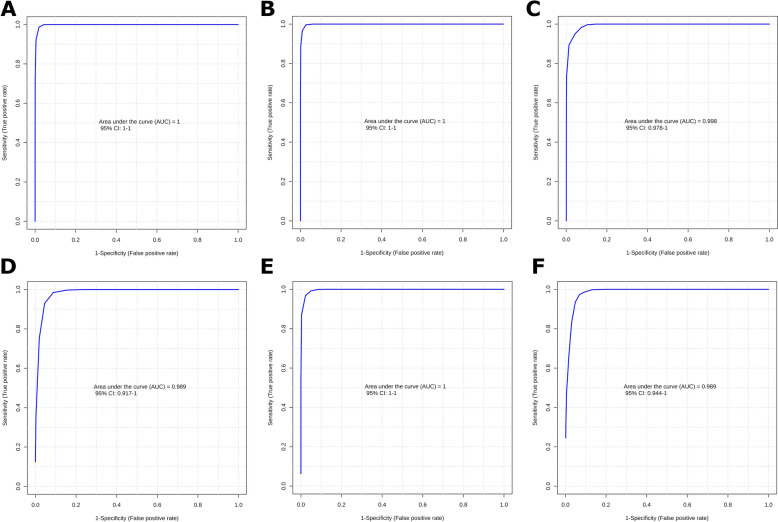
**Area Under the Receiver Operating Characteristic Curve (AUC-ROC) estimated through fivefold cross-validation.** **A** TB versus HC, **B** TB versus PTB, and **C** PTB versus HC acquired by high-resolution NMR, and **D** TB versus HC, **E** TB versus PTB, and **F** PTB versus HC acquired by benchtop NMR spectroscopy. TB: tuberculosis infection. PTB: paratuberculosis infection. HC: healthy control.

For HR-NMR (Figures 3A–C), the comparison between TB and HC groups, based on the concentration of 12 metabolites, yielded a perfect AUC-ROC of 1.0 [95% confidence interval (CI): 1–1], demonstrating 100% sensitivity and specificity. Similarly, the TB versus PTB comparison, considering 10 metabolites, also achieved an AUC-ROC of 1.0 (95% CI: 1–1), again with 100% sensitivity and specificity. In contrast, the PTB versus HC comparison, using 6 metabolites, exhibited a slightly lower AUC-ROC of 0.998 (95% CI: 0.978–1), with 100% sensitivity and 92% specificity.

For b-NMR (Figures 3D-F), the comparison between TB and HC, considering 6 metabolites, achieved a strong AUC-ROC of 0.989 (CI 95%: 0.917–1), with 100% sensitivity and 88% specificity. The comparison between TB and PTB, using 3 metabolites, reached a perfect AUC-ROC of 1.0 (CI 95%: 1–1), showing 100% sensitivity and specificity. Lastly, the comparison of PTB and HC, based on 6 metabolites, resulted in an AUC-ROC of 0.989 (CI 95%: 0.944–1), with 100% sensitivity and 96% specificity.

### Metabolic pathway analysis

**Figure 4:**
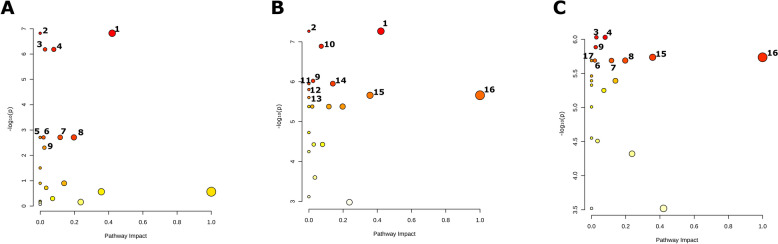
**Metabolic pathways analysis comparing (A) TB versus HC, (B) TB versus PTB, and (C) PTB versus HC**. The Y-axis represents the statistical *p*-values obtained from pathway enrichment analysis, while the X-axis shows the pathway impact values derived from pathway topology analysis. The node size is proportional to the pathway impact, and the color scale indicates significance, ranging from highly significant (*p* < 0.001, red) to less significant (*p* ≈ 0.05, yellow). Pathway assignments: 1, Starch and sucrose metabolism; 2, Glycolysis/Gluconeogenesis; 3, Pyruvate metabolism; 4, Glutathione metabolism; 5, Arginine biosynthesis; 6, Alanine, aspartate and glutamate metabolism; 7, Arginine and proline metabolism; 8, Glycine, serine and threonine metabolism; 9, Tyrosine metabolism; 10, Phenylalanine metabolism; 11, Phenylalanine, tyrosine and tryptophan biosynthesis; 12, Glycerolipid metabolism. TB = Tuberculosis infection. PTB = Paratuberculosis infection. HC = Healthy Control.

A metabolic pathway analysis was conducted to identify the primary significant pathways implicated in the pathogenesis of TB and PTB (Figure 4). Pathway and enrichment analyses conduced on the 14 metabolites detecting with HR-NMR, with statistical significance considered at *p*-value < 0.01.

Comparative metabolomic analyses revealed distinct metabolic profiles associated with each group. The pathways involved included of glycolysis and pyruvate metabolism (involving high levels of acetate and glucose, and lower levels of lactate in TB group), alanine, aspartate and glutamate metabolism (linked to high levels of glutamate in TB group), and, arginine and proline metabolism (creatine and glutamate are involved). These pathways were implicated in all three comparations; TB vs. HC, TB vs. PTB and PTB vs. HC.

Additional metabolic pathways were identified as being implicated in PTB vs. TB and PTB vs. HC comparisons, including glycine, serine and threonine metabolism (involving betaine and creatine), tyrosine metabolism (implicating tyrosine), the biosynthesis of phenylalanine, tyrosine and tryptophan and phenylalanine metabolism (involving phenylalanine and tyrosine), phenylalanine metabolism (implicating phenylalanine and tyrosine) and glycerolipid metabolism (involving glycerol), all of which exhibited low levels of the metabolites involved in the PTB group.

## Discussion

Our study demonstrated the ability of both HR-NMR and b-NMR to differentiate TB-infected, PTB-infected, and healthy control goats with high diagnostic accuracy. This finding reinforces the growing body of evidence supporting NMR-based metabolomics as a reliable approach for infection profiling, in line with previous studies conducted in bovine [10, 38] and human TB diagnostics [20, 21]. The classification models developed here yielded excellent predictive performance, with cross-validation confirming near-perfect AUC-ROC values across all pairwise comparisons. Notably, both platforms identified key metabolites associated with *Mycobacterium* infection—14 in HR-NMR and 8 in b-NMR—highlighting consistent metabolic signatures despite differences in spectral resolution. While HR-NMR offers superior sensitivity and shorter acquisition times, the results obtained with b-NMR emphasize its potential as a more accessible, cost-effective, and robust alternative for routine screening, particularly in field or veterinary settings where high-field instruments are not available. Nevertheless, further studies including larger cohorts and longitudinal follow-up are warranted to validate the stability and clinical translation of these metabolic biomarkers.

In particular, the differentiation of serum metabolic profiles revealed key metabolites involved in carbon and nitrogen metabolism—critical pathways for *Mycobacterium tuberculosis* survival and virulence [39, 40]. Among the metabolites identified, acetate and glycerol, as carbon sources, were significantly elevated in TB-infected goats. These metabolites play essential roles in the bacterium’s energy metabolism [41]. The increase in glucose levels observed in TB-infected goats likely reflects the heightened glycolytic demands of activated macrophages [42], while the reduction in lactate points toward a shift from anaerobic to aerobic metabolism in TB infection [43, 44]. In this line, lower levels of 3-hydroxybutyrate, a ketone body, indicate impaired lipid metabolism, which is consistent with findings observed in TB patients [45]. It is also synthesized from acetoacetate in the liver, particularly during fasting condition [39].

The observed changes in amino acids, including elevated glutamate, leucine, threonine, and tyrosine, further underscore the role of nitrogen metabolism in TB pathogenesis [46, 47]. The elevated levels of lysine and phenylalanine observed in infected goats are attributed to the involvement of these amino acids in immune cell proliferation and mitosis and the expansion process of T and B lymphocytes following activation by bacterial antigen [48, 49]. Glucose and glutamine serve as essential metabolites in the metabolic reprogramming process that drives macrophage activation toward the M1 phenotype [50]. Glutamate serves as both a product of glutamine metabolism and a precursor for the de novo synthesis of glutathione. Glutathione plays a critical role in mitigating elevated reactive oxygen species (ROS) levels directly or indirectly (being involved in the transport of nitric oxide), resulting in the antibacterial function of macrophages [39, 51, 52]. It contributes to the redox homeostasis of M1 macrophages by directly serving as a substrate for glutathione synthesis or indirectly facilitating cytidine uptake through coupling with the xCT antiporter [50].

Finally, creatine is synthesized from arginine, and increased creatine production may lead to a decrease of the intracellular availability of arginine for antibacterial nitric oxide production. However, endogenous creatine synthesis primarily occurs in the kidneys and liver [52]. Creatinine, a metabolic byproduct of creatine, may be involved in the synthesis of nitrogenous compounds [21]. Furthermore, betaine, which is a product of choline oxidation, exhibits osmoprotective properties, and it has been demonstrated that mycobacteria can accumulate it in response to osmotic stress [53].

PTB is another *Mycobacterium*-induced infection that can be effectively detected using both HR-NMR and b-NMR platforms. Previous metabolomic studies have reported alterations in fatty acids and other lipid-related metabolites, reflecting changes in lipid biosynthesis and rumen microbiota activity [54, 55]. In contrast, our observations regarding creatine and creatinine levels differ from those described in previous studies conducted in cattle [56–58]. These interspecies discrepancies may stem from differences in diet and physiology, as well as from the metabolic relationship between creatinine and arginine, the latter being essential for T-lymphocyte proliferation. Additionally, metabolites such as phenylalanine and tyrosine are downregulated in PTB infections owing to alteration in the degradation and biosynthesis of ketonic bodies [59]. A reduction in glutamate levels was also observed, likely reflecting its involvement in glutathione metabolism, where it contributes to antioxidant defense, immune regulation, and gastrointestinal function [57].

A limitation of this study is the need for larger-scale investigations to confirm these findings and further assess diagnostic specificity, particularly in the presence of co-infections that may influence metabolic profiles. Nevertheless, our positive results with b-NMR add to previous successful applications in bovine TB, suggesting the technique’s adaptability to other domestic and wild species where conventional diagnostics, such as IGRA and SIT tests, are impractical. Notably, b-NMR, despite its lower sensitivity and resolution compared with HR-NMR, detected significant metabolic changes across all groups, reinforcing its value as a more accessible, cost-effective diagnostic alternative. The ability of b-NMR to differentiate TB from PTB is especially noteworthy, as PTB can compromise the specificity of traditional TB diagnostics. This underscores b-NMR’s potential not only as a reliable tool in TB diagnostics for livestock but also as a possible method for PTB detection, offering a promising addition to current ante mortem diagnostic options.

In conclusion, this study provides promising evidence for the application of NMR-based metabolomics, including the use of benchtop instruments, in the diagnosis of TB. Our findings indicate that b-NMR could serve in the future as a practical and effective tool in clinical settings, particularly in regions where access to high-resolution systems may be limited. The identification of metabolomic fingerprints associated with TB and PTB offers a promising avenue for further research and clinical application, potentially improving the accuracy and accessibility of TB diagnostics in veterinary and human medicine.

## Data Availability

The serum spectra data presented in this article have been deposited in a recognized data repository (Figshare) with a digital object identifier, and will be made publicly available upon publication of the article [60].
